# To gel or not to gel: differential expression of carrageenan-related genes between the gametophyte and tetasporophyte life cycle stages of the red alga *Chondrus crispus*

**DOI:** 10.1038/s41598-020-67728-6

**Published:** 2020-07-13

**Authors:** Agnieszka P. Lipinska, Jonas Collén, Stacy A. Krueger-Hadfield, Theo Mora, Elizabeth Ficko-Blean

**Affiliations:** 1 Station Biologique de Roscoff, UMR8227, Laboratory of Integrative Biology of Marine Models, CNRS and Sorbonne University, Place Georges Teissier, 29688 Roscoff, France; 2 Department of Biology, The University of Alabama At Birmingham, Campbell Hall 464, 1300 University Blvd, Birmingham, AL 35294 USA

**Keywords:** Glycobiology, Differentiation, Ecological genetics, Cell wall, Gene expression, Development

## Abstract

*Chondrus crispus* is a marine red alga with sulfated galactans, called carrageenans, in its extracellular matrix. *Chondrus* has a complex haplodiplontic life cycle, alternating between male and female gametophytes (n) and tetrasporophytes (2n). The *Chondrus* life cycle stages are isomorphic; however, a major phenotypic difference is that carrageenan composition varies significantly between the tetrasporophytes (mainly lambda-carrageenan) and the gametophytes (mainly kappa/iota-carrageenans). The disparity in carrageenan structures, which confer different chemical properties, strongly suggests differential regulation of carrageenan-active genes between the phases of the *Chondrus* life cycles. We used a combination of taxonomy, biochemistry and molecular biology to characterize the tetrasporophytes and male and female gametophytes from *Chondrus* individuals isolated from the rocky seashore off the northern coast of France. Transcriptomic analyses reveal differential gene expression of genes encoding several galactose-sulfurylases, carbohydrate-sulfotransferases, glycosyltransferases, and one family 16 glycoside hydrolase. Differential expression of carrageenan-related genes was found primarily between gametophytes and tetrasporophytes, but also between the male and female gametophytes. The differential expression of these multigenic genes provides a rare glimpse into cell wall biosynthesis in algae. Furthermore, it strongly supports that carrageenan metabolism holds an important role in the physiological differentiation between the isomorphic life cycle stages of *Chondrus*.

## Introduction

Red algae are ancient eukaryotic organisms, providing the first clearly identified fossil record of eukaryotic photosynthetic life and the earliest known extant form of complex multicellularity^1–3^. A prerequisite for complex multicellularity is the development of an extracellular matrix (ECM). While cellulose is present in red algae, the major ECM components feature specific and often negatively charged (mainly sulfated) polysaccharides^4^. Intuitively, this feature may be associated with the algal need of a resilient highly hydrated matrix to cope with their specific environment in sea water and to resist the extreme changes of environmental conditions driven by tidal cycles. Furthermore, the micro-heterogeneity of the sulfations on the glycans can have structural or functional roles, such as in signalling or pathogen defense^5,6^.

**Figure 1:**
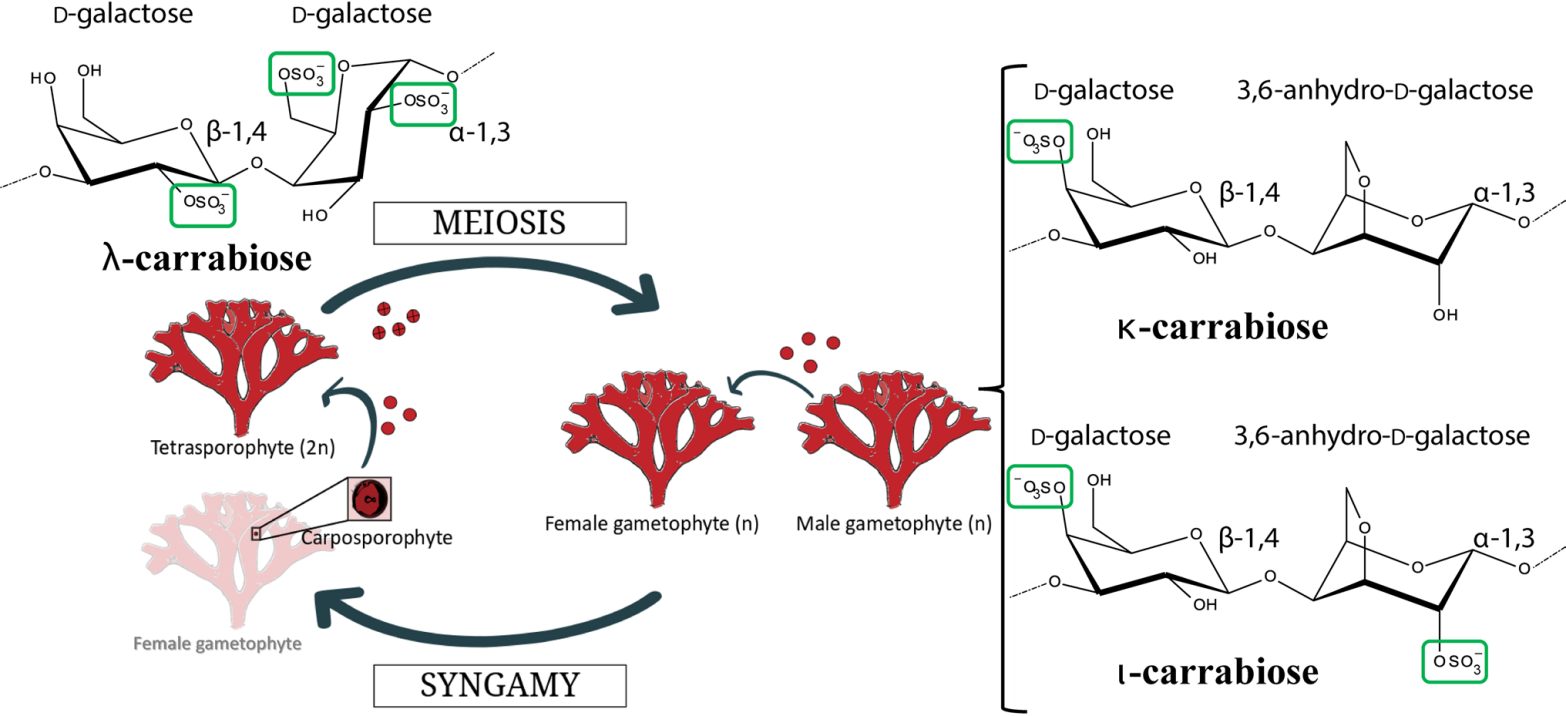
The isomorphic life cycle stages of *Chondrus crispus* and their dominant carrabiose motifs. The life cycle consists of an alternation between haploid dioecious gametophytes and a diploid tetrasporophyte. After fertilization, the zygote develops within the carposporophyte on the female gametophyte and is mitotically amplified—producing thousands of diploid carpospores that after release will give rise to tetrasporophytes. These carrabiose structures represent the dominant repeating disaccharides found in the *Chondrus* ECM. Natural carrageenans are hybrid polymers of repeating units, for example *Chondrus* gametophytes are composed primarily of kappa- and iota-carrageenan motifs (~ 70% and 20%) and the non-cyclized mu- and nu-carrageenan motifs (~ 8% and 2%)^19,20^.

Carrageenophyte red algae produce carrageenan, a linear sulfated galactan with alternating α-1,3-/β-1,4-linkages, in their ECMs. Some carrageenan motifs contain the unique red algal bicyclic sugar 3,6-anhydro-D-galactose produced by the action of galactose-sulfurylase enzymes^7^. The 3,6-anhydro-D-galactose moiety is present in kappa- and iota-carrabiose motifs, -[D-galactose-4-sulfate-(β-1,4)-3,6-anhydro-D-galactose-(α-1,3)]- and -[D-galactose-4-sulfate-(β-1,4)-3,6-anhydro-D-galactose-2-sulfate-(α-1,3)]-, respectively, and not in the lambda carrabiose motif, -[D-galactose-4-sulfate-(β-1,4)-D-galactose-2,6-disulfate-(α-1,3)]- (Fig. 1). Further differentiating the carrabiose structures are the sulfate groups: kappa-carrabiose has one sulfate group, iota-carrabiose has two sulfate groups and lambda carrabiose has three sulfate groups. However, not all red algae are carrageenophytes. Agarophyte red algae have varying proportions of porphyranobiose, -[D-galactose-(β-1,4)-L-galactose-6-sulfate-(α-1,3)]-, and agarobiose, -[D-galactose-(β-1,4)-3,6-anhydro-L-galactose-(α-1,3)]-, in their ECMs^4,8^. Contradictorily, there are reportedly carrageenophyte red algae that are able to produce minor to significant amounts of sulfated agars and DL-galactan hybrids^9,10^ and agarophyte red algae that have DL-galactan hybrid structures^11,12^. Overall, it is not fully understood how the differences in galactan structures arose between the carrageenophyte and agarophyte red algae.

**Figure 2:**
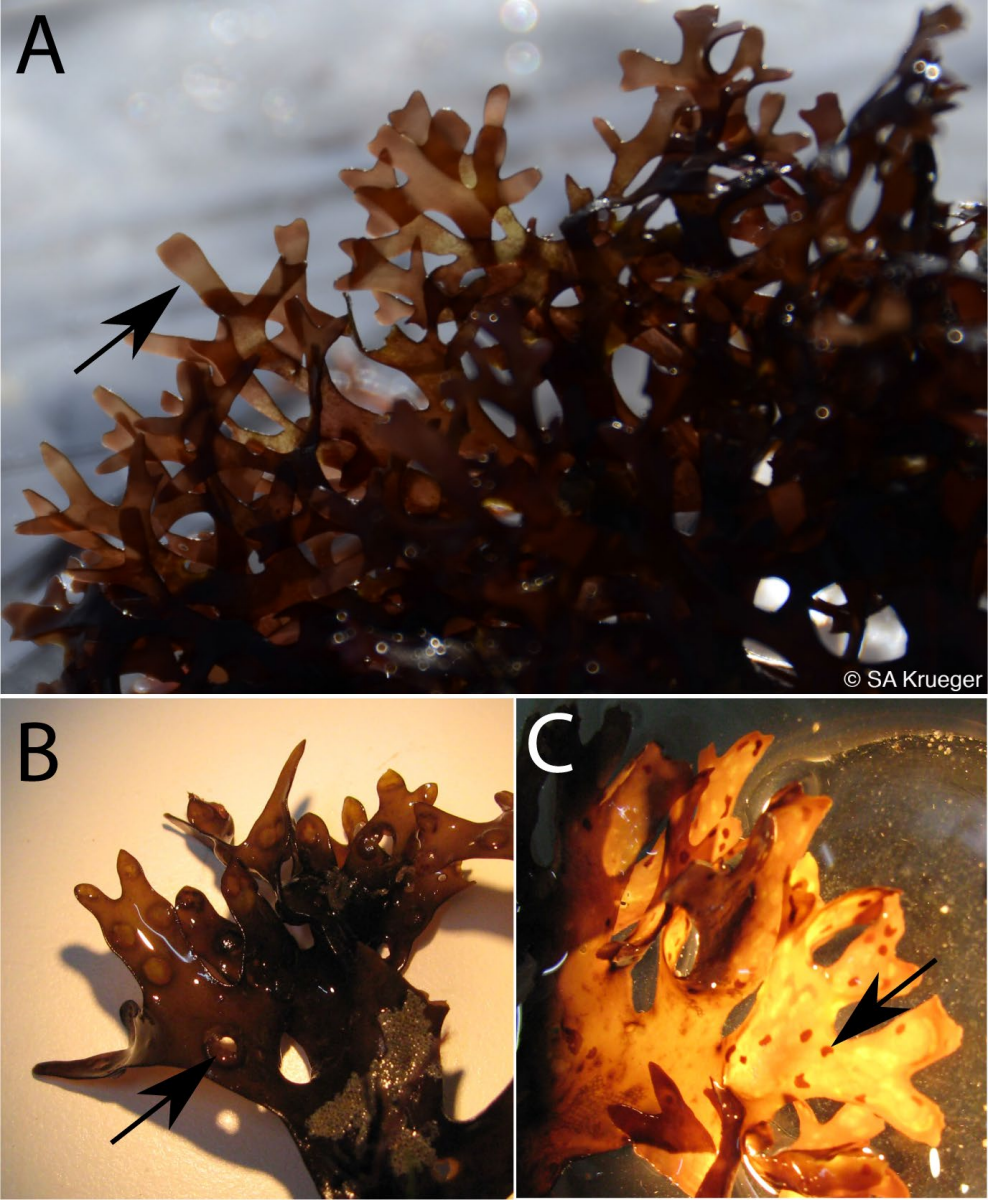
*Chondrus* life cycle stages may be assessed through presence of reproductive structures. (**A**) Reproductive male gametophyte identified by a pinkish to white band of spermatangial shown by a black arrow^35^, (**B**) Fertilized female gametophytes identified by the presence of cystocarps shown by a black arrow, and (**C**) The presence of tetrasporangial sori are shown by a black arrow on reproductive tetrasporophytes^36^. Photo credit SA Krueger-Hadfield.

*Chondrus crispus*, or Irish moss, is a carrageenophyte red alga (Rhodophyta) that grows on the rocky shores of the North Atlantic Ocean. *Chondrus* has a complex, haplodiplontic life cycle (Fig. 1), alternating between male and female gametophytes (n) and tetrasporophytes (2n); further complexity in the life cycle is demonstrated by zygote-derived carposporophytes (2n) that are attached to the female gametophyte^13,14^ (Fig. 2). The male and female gametophytes and the tetrasporophytes are isomorphic and are difficult to distinguish in the absence of reproductive structures. However, an interesting observation is that the ECMs of *Chondrus* tetrasporophytes are lambda-carrageenan dominant whereas gametophytes are kappa-/iota-carrageenan dominant^15–20^; this difference is in part why *Chondrus* ploidy ratios are so well known across the North Atlantic^21^.

Different carrageenan composition, which has an effect on the physiological states of gametophytes and tetrasporophytes, has important implications in the ecology and evolution of *Chondrus* species. For example, *Chondrus* tetrasporophytes are susceptible to infection by the green algal pathogen *Ulvella (Acrochaete) operculata*^22,23^. Gametophytes are resistant to infection, but if lambda-carrageenan oligosaccharides are artificially introduced to the milieu the pathogen produces specific polypeptides and there is a marked increase in virulence^6^. Conversely, the green algal pathogen demonstrates significantly reduced virulence on the susceptible tetrasporophytes when treated with kappa-carrageenan oligosaccharides^6^. The differences in susceptibility to *U. operculata* has a direct correlation to carrageenan metabolites and shows that the carrageenans specific to the life cycle stage may demonstrate functionally distinct biological signalling properties. The tetrasporophyte susceptibility to infection could also scale up to population-level processes. Indeed, many *Chondrus* populations are gametophyte-biased. When infection rates were compared with ploidy ratios in natural populations in northwestern France^14^, they were significantly higher in low shore and denser populations that were correspondingly gametophyte-biased. In contrast, on the high shore, where populations are less dense, infections levels were lower and the high shore population was not different from the expected ratio of $\sqrt{2}$:1, due to differential reproduction by females and tetrasporophytes^24,25^.

**Figure 3:**
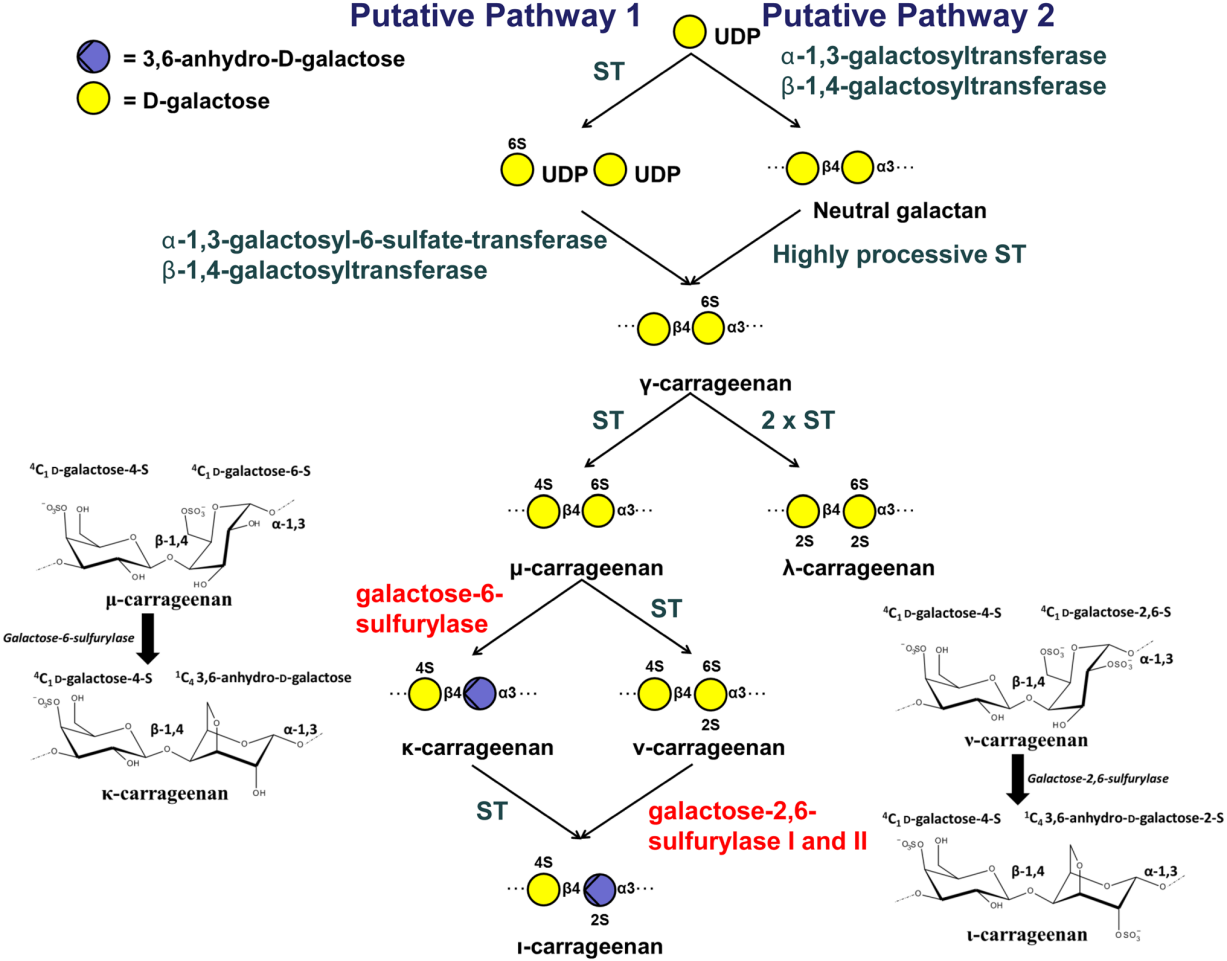
The putative carrageenan biosynthetic pathways in red algae^26,27^*.* Steps that have been experimentally elucidated are highlighted in red^7,28^. Galactose-sulfurylase reactions that have been experimentally elucidated are shown in more detail, that is the conversion of mu- to kappa-, and nu- to iota-carrageenan^7,28^. ST = sulfotransferase.

A pathway for carrageenan biosynthesis was first proposed in 1979^26^ and updated in 2015^27^. To date, the only red algal enzymes biochemically characterized in this pathway are the galactose-sulfurylases, which catalyse the formation of 3,6-anhydro-galactose through removal of the C6 sulfate group of galactose (Fig. 3)^7,28^. These enzymes are unique to red algae and are involved in the last step of carrageenan biosynthesis, controlling the conformation of the polysaccharide chain. Formation of the 3,6-anhydro-bridge from the precursors substrates α-D-galactose-6-sulfate or α-D-galactose-2,6-disulfate to the products α-3,6-anhydro-D-galactose or α-3,6-anhydro-D-galactose-2-sulfate, respectively, results in a ring flip from ^4^C_1_ to ^1^C_4_ and increases the hydrophobic nature of the monosaccharide moieties. As a tertiary structural consequence, the 3,6-anhydro-bridges promote formation of helices in the carrageenan chain, which then form gel-networks^29^. The in vitro rheological properties of the carrageenans differ depending on the degree of sulfation and presence of 3,6-anhydro-D-galactose. Industry exploits kappa- and iota-carrageenans for their gel forming properties while lambda-carrageenan is used mainly as a thickening agent^30^. Little is known on the in vivo rheological properties of the different carrageenans within the ECM though gametophyte distal tissues are stronger, more extensible and stiffer than tetrasporophyte distal tissues^31^. No biomechanical differences were found in the stipe holdfast junctions^31^, suggesting neither gametophytes nor tetrasporophytes have an advantage relative to one another against wave exposure. The molecular composition of the carrageenan metabolites in the ECM is structurally distinct between the life cycle stages, and yet morphologically the *Chondrus* individuals resemble one another texturally and visually. To the best of our knowledge there are no studies describing differences in carrageenan structure between male and female gametophytes though it would not be unexpected to find molecular differences. In any case, there is strong reason to believe that carrageenan structure plays a role in physiological differentiation during the life cycle of *Chondrus*.

The differences in carrageenan composition between the *Chondrus* gametophytes and tetrasporophytes^15–18^ suggest that there is differential gene expression of carrageenan-related genes between the life cycles stages. Furthermore, the maintenance of isomorphic haplodiplontic life cycles, such as found in *Chondrus,* is predicted if the two stages exploit different niches, even if those differences are subtle^32^. Differential gene expression of genes so integral to the structure of the thallus would fit the predictions^32^, but studies explicitly testing this hypothesis are rare^33^. With the available genomic and genetic resources and the accessibility of the different life stages in the field^34^, *Chondrus* provides an ideal model to address the gene expression in its isomorphic gametophyte and tetrasporophyte stages. Here, we use a combination of taxonomy, biochemistry, molecular biology and transcriptomics to identify select carrageenan-related enzymes that contribute to the physiological differentiation of the ECMs between the life cycle stages and these results are put in the context of the predicted carrageenan biosynthesis pathway^26,27^.

## Materials and methods

### Sample collection

Three male gametophytes (n), three female gametophytes (n) and three tetrasporophytes (2n) of *Chondrus crispus* were collected in Roscoff, France at the Pointe de Bloscon (48.726° N—3.969° W) on August 16, 2018 just after low tide (Table 1). The tissue was cleaned briefly in sterile sea water and padded dry using paper towels. Life cycle stage was initially assessed through presence of reproductive structures (Fig. 2). Female gametophytes (haploid), male gametophytes (haploid) and tetrasporophytes (diploid) were identified by their reproductive organs: (i) fertilized female gametophytes were identified by the presence of cystocarps, (ii) reproductive male gametophytes were identified by a pinkish to white band of spermatangial sori 3 to 10 mm below the apex^35^ and (iii) reproductive tetrasporophytes were identified by the presence of tetrasporangial sori^36^. Carposporophytes, the reproductive structures that remain on the female gametophyte post-fertilization in which the females nurture developing zygotes, were carefully excised from the female gametophytes. Similarly, sori containing tissue was removed from the male gametophyte and tetrasporophyte material. Samples were cleaned with sterile sea water, patted to remove excess liquid and flash frozen in liquid nitrogen on-site for later use.

**Table 1:** Samples collected, ploidy, sex, library reference and accession numbers.

Species	Sample name	Sex	Library reference	No. of reads	Accession no
*Chondrus crispus*	F1	Female gametophyte	ADPH-67	36,776,986	SAMN14087152
	F2	Female gametophyte	ADPH-68	35,416,293	SAMN14087153
	F3	Female gametophyte	ADPH-69	34,679,722	SAMN14087154
	M1	Male gametophyte	ADPH-70	39,089,179	SAMN14087155
	M2	Male gametophyte	ADPH-71	43,860,997	SAMN14087156
	M3	Male gametophyte	ADPH-72	44,533,481	SAMN14087157
	T1	Tetrasporophyte	ADPH-74	39,071,297	SAMN14087158
	T2	Tetrasporophyte	ADPH-76	35,706,334	SAMN14087159
	T3	Tetrasporophyte	ADPH-77	37,034,377	SAMN14087160

### Resorcinol test

The resorcinol test was performed to provide colourimetric differentiation of tetrasporophyte tissue relative to gametophyte tissue^14,36–38^. A 13.6 mM stock resorcinol solution was prepared by diluting 30 mg of powdered resorcinol in 20 mL of distilled water (stored in the dark). The acetal stock solution was made by adding 8 μL of acetal (1,1-diethoxyethane) to 1992 μL water. The reactive resorcinol-acetal solution was made right before usage, 25 mL of concentrated HCl (37%, commercial) was added to 2.25 mL of the stock resorcinol solution followed by addition of 250 μL of acetal stock solution. Small fragments of the algae, approximately 1 mm × 1 mm, were placed in 1.5 mL Eppendorf tubes then 500 μL of the reactive resorcinol-acetal solution was added. The tubes were incubated for 10 min at 80 °C and then cooled on ice for two minutes followed by qualitative colourimetric evaluation of the staining. Gametophytes result in positive tests and turn the solution a reddish colour, while tetrasporophytes result in a negative test (Table 2, Supplementary Fig. 1).

**Table 2:** Results of the ploidy test using microsatellite markers for 6 genotyped loci for the *Chondrus* samples. (Chc24_VIC, Chc31_NED, Chc40_6FAM, Chc02_VIC, Chc03_6FAM, Chc04_PET; from^14,36^) and from the resorcinol assay.

Sample name	Sex	Resorcinol test	Number of heterozygous loci out of 6 genotyped loci
F1	Female gametophyte	Positive	0
F2	Female gametophyte	Positive	0
F3	Female gametophyte	Positive	0
M1	Male gametophyte	Positive	0
M2	Male gametophyte	Positive	0
M3	Male gametophyte	Positive	0
T1	Tetrasporophyte	Negative	6
T2	Tetrasporophyte	Negative	5
T3	Tetrasporophyte	Negative	5

### Microsatellite analyses

Total genomic DNA was extracted using Machery-Nagel Plant DNA kit, following manufacturers instructions. We used the microsatellite loci Chc_24 (flourescent label VIC), Chc_31 (flourescent label NED), and Chc_40 (flourescent label 6-FAM) from^36^ and Chc_02 (flourescent label VIC), Chc_03 (flourescent label 6-FAM), and Chc_04 (flourescent label PET) from^14^. Simplex PCRs were performed using either a SimpliAmp or ProFlex thermocycler (Applied Biosystems, Foster City, CA, USA): 20 μL final volume, 5 μL of DNA template, 1 × buffer, 250 μM dNTP, 2 mM MgCl_2_, 300 nM flourescently labeled forward primer, 100 nM unlabeled forward primer, and 400 nM of unlabeled reverse primer, and 0.5 U taq polymerase (GoTaq Flexi, Promega). The PCR program included: 5 min at 95 °C, followed by 30 cycles of 30 s at 95 °C, 30 s at either 60 °C (Chc_24, Chc_31, Chc_40) or 55 °C (Chc_02, Chc_03, Chc_04), 30 s at 72 °C; and finally, 10 min at 72 °C. One µL of each PCR product was poolplexed into Poolplex 1 (Chc_23, Chc_24, Chc_31, Chc_40) and Poolplex 2 (Chc_02, Chc_03, Chc_04) and 10 µL of loading buffer containing 0.3 µL of size standard (GeneScan – 500 Liz, Applied Biosystems, Foster City, CA, USA) plus 9.7 µL of Hi-Di formamide (Applied Biosystems, Foster City, CA, USA). Samples were electrophoresed on an ABI 3730xL genetic analyzer equipped with a 96-caipllary array (Applied Biosystems, Foster City, CA, USA). Alleles were scored manually using GENEIOUS PRIME 2019.0.3 (https://www.geneious.com). Multilocus genotypes were then compared to the results from the resorcinol tests to verify all gametophytes displayed one allele per locus and tetrasporophytes displayed one or two alleles per locus (i.e., homozygotes or heterozygotes).

### RNA extractions

RNA of the nine *Chondrus* thalli was extracted using a the Qiagen RNeasy Plant minikit and following the manufacturer’s protocol. The frozen samples were transferred to 1.5 mL Eppendorf tubes and ground down in liquid nitrogen using a pestle to obtain a fine powder. Lysis buffer (RLT) with 0.04 M DTT (inhibitor of RNases) was added immediately after grinding. The lysates were centrifuged and the supernatants were recovered in new tubes. Ethanol was added to 0.5 V and the samples were loaded over two loads onto RNeasy Mini spin columns which was followed by several wash steps. RNA was eluted with 30 μL RNase free water. Quality and quantity of the samples were determined by measuring the A_260_/A_280_ and using a Qubit 2.0 Fluorometer with the Qubit RNA BR assay kit.

### Transcriptomic analysis

Between 800 ng–1 µg of total RNA per sample was sent for sequencing using Illumina HiSeq 4,000 technology at Fasteris S.A. company (Plan-les-Ouates, Switzerland). Single-end 150 bp RNA reads were trimmed of adapter sequences and low quality bases using Trimmomatic v0.33 (LEADING:3 TRAILING:3 SLIDINGWINDOW:4:15 MINLEN:50)^39^. Cleaned RNAseq reads were used first to ameliorate gene model predictions, incorporating the previous annotations^40^, with PASA v2.3.3 pipeline^41^. The improved gene models (see Supplementary File 1) were than used as a reference to estimate expression (TPM) and number of reads per gene using Kallisto (v0.44.0) with 1,000 bootstraps^42^. The number of reads per gene was used to compare the differential gene expression between the three life phases (Male vs Females, Males vs Tetrasporophytes, Females vs Tetrasporophytes) using DESeq2 package in R Studio^43^. Genes were considered to be deferentially expressed if they exhibited at least a twofold difference in expression (FC >  = 2) between investigated samples and a false discovery rate (FDR) of < 0.05. The data has been deposited in the SRA database under BioProject ID PRJNA606187. The accession numbers for the raw sequence data are provided in Table 1.

### Glycoside hydrolase (GH), glycosyl transferase (GT), galactose-sulfurylase and sulftotransferase (ST) annotations

Enzymes were first identified by homology with a subset of selected enzymes from each CAZyme (Carbohydrate-active enzyme), sulfurylase or sulfotransferase databank using the local BLAST function with an E value threshold of 1.0E^-4^ in the program ngKLAST (Korilog). Initial annotations were further verified by BLASTp^44^ searches against the UniProtKB/SWISSPROT database, signature scans using InterPro^45^, and HMMER searches against the Pfam database^46^. Whenever possible, each enzyme was assigned to a family.

### Phylogeny

Galactose-sulfurylase homologous sequences identified from *Chondrus* were selected by Blastp at NCBI^47^. The proteins were aligned using MAFFT^48^ with the L-INS-I algorithm and the scoring matrix Blosum62. The alignment was manually refined using BioEdit^49^. The evolutionary history was inferred by using the Maximum Likelihood method and Whelan And Goldman model^50^. The tree with the highest log likelihood (−5,187.58) is shown. The percentage of trees in which the associated taxa clustered together is shown next to the branches. Initial tree(s) for the heuristic search were obtained automatically by applying Neighbor-Join and BioNJ algorithms to a matrix of pairwise distances estimated using a JTT model, and then selecting the topology with superior log likelihood value. A discrete Gamma distribution was used to model evolutionary rate differences among sites (5 categories (+ G, parameter = 2.1008)). The tree is drawn to scale, with branch lengths measured in the number of substitutions per site. This analysis involved 16 amino acid sequences. There were a total of 263 positions in the final dataset. Evolutionary analyses were conducted using MEGA X^51^.

## Results and discussion

### Assessment of life cycle stage

The nine *Chondrus* thalli used in this study were sampled from the rocky intertidal and a morphological determination of ploidy and sex was made in the field based on the presence of reproductive structures (Fig. 2). Gametophytes were also differentiated from the tetrasporophytes by their iridescence, which the tetrasporophytes lack^15^.

To further support the ploidy assessment of the *Chondrus* individuals we performed the resorcinol test^37,38^. Previous work has shown this test to be reasonably accurate in determining ploidy stage when accompanied by microsatellite genotyping that can distinguish haploid from diploid thalli^14,36^. The colourimetric assay was positive for the male and female gametophytes and negative for the tetrasporophytes supporting the initial morphological assessment of life cycle stage (Table 2, Supplementary Fig. 1).

Microsatellite multilocus genotyping also confirmed ploidy though none of the microsatellites are sex-specific or have sex-specific alleles. All gametophytes exhibited a single allele per locus, whereas the tetrasporophytes had two alleles at five of the six loci used to genotype the thalli (Table 3). At the Port de Bloscon, tetrasporophytes had, on average, 3.9 heterozygous loci out of the same 6 loci genotyped both determined previously^36^ and in this study.

**Table 3:** Microsatellite results for the *Chondrus* samples, sizes of the PCR fragments are shown in base pairs.

Sample ID	Chc24_VIC		Chc31_NED		Chc40_6FAM		Chc02_VIC		Chc03_6FAM		Chc04_PET	
F1	194	194	257	257	169	169	278	278	253	253	292	292
F2	184	184	257	257	169	169	278	278	253	253	292	292
F3	178	178	217	217	179	179	280	280	0	0	298	298
M1	194	194	213	213	159	159	260	260	253	253	294	294
M2	178	178	261	261	169	169	260	260	263	263	292	292
M3	192	192	213	213	161	161	260	260	283	283	318	318
T1	172	186	211	215	189	225	280	280	249	255	292	314
T2	212	226	209	217	193	197	244	254	239	249	262	272
T3	172	222	207	213	181	181	278	280	249	253	294	298

### Carrageenan biosynthesis

#### Galactose-sulfurylases

**Figure 4:**
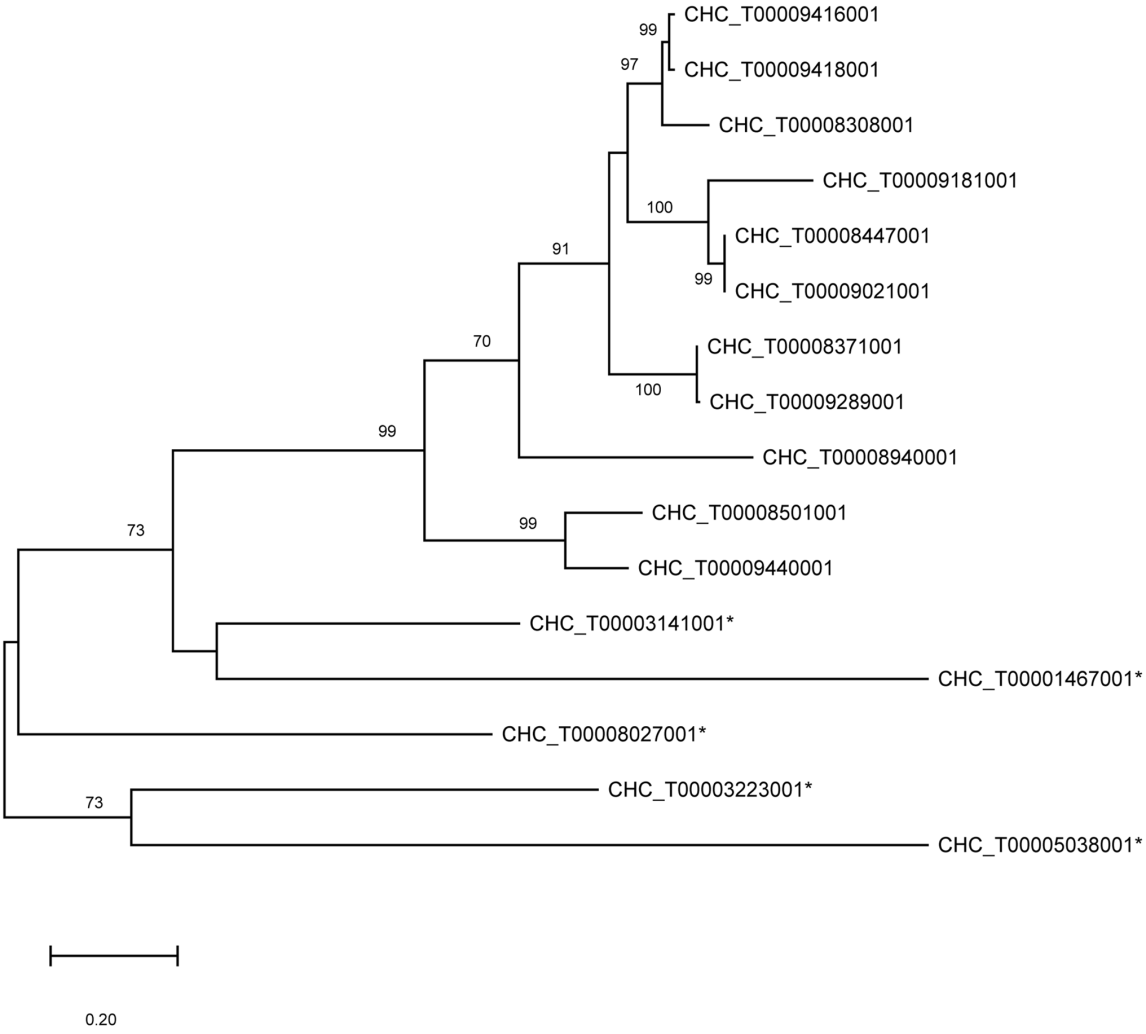
Phylogenetic analysis on the galactose-sulfurylase II family in *Chondrus*. The evolutionary history was inferred by using the Maximum Likelihood method and Whelan And Goldman model^50^. The unrooted phylogenetic tree with the highest log likelihood (−5,187.58) is shown. The tree is drawn to scale, with branch lengths measured in the number of substitutions per site. This analysis involved 16 amino acid sequences. There were a total of 263 positions in the final dataset. Bootstraps above 70 are shown. Evolutionary analyses were conducted in MEGA X^51^. The newly identified putative galactose-sulfurylase enzymes are marked with an asterisk.

The final step of iota-carrageenan biosynthesis in *Chondrus*, namely the conversion of nu- carrageenan to iota-carrageenan (Fig. 3), has been clearly shown in *Chondrus* gametophytes through the biochemical characterization of purified wild type D-galactose-2,6-sulfurylase I and II^7^. There is only one copy of the galactose-2,6-sulfurylase I gene encoded in the *Chondrus* genome; however, the galactose-sulfurylase II enzymes are one of the rare multigenic families in *Chondrus*^40^. During the annotation of the differentially expressed genes from *Chondrus* we identified five new putative galactose-sulfurylase II enzymes bringing the total number of *Chondrus* galactose-sulfurylase II enzymes from 11^40^ to 16 (Table 4). An updated phylogenetic analysis on the galactose-sulfurylase II family in *Chondrus* is shown in an unrooted phylogenetic tree in Fig. 4. The newly identified galactose-sulfurylase II proteins in *Chondrus* are phylogenetically distant leading us to speculate on differing substrate specificities within the galactose-sulfurylase II family. For example, D-galactose-6-sulfurylase activity, for the enzymatic transformation of mu- carrageenan to kappa- carrageenan (Fig. 3), has been detected in *Chondrus* extracts^28^ though the enzyme(s) have not been identified.

**Table 4:** Annotated carrageenan-biosynthesis genes. Averaged TPMs are shown for the *Chondrus* female and male gametophytes and the tetrasporophytes. The differential gene expression is shown for the pairwise comparisons between the life cycle stages. Significant differences are marked with an asterisk. F is female gametophyte, M is male gametophyte, and T is tetrasporophyte.

Gene	Annotation	Gene expression level (TPM)			Differential expression (log2FoldChange)		
		F	M	T	M/F	T/F	T/M
CHC_T00001467001	CcGal-Sulfurylase II	0.34	0.19	572.93	−1.11	10.22*	11.31*
CHC_T00003141001	CcGal-Sulfurylase II	7.75	6.30	237.17	−0.56	4.41*	4.98*
CHC_T00003223001	CcGal-Sulfurylase II	20.88	38.22	96.57	0.66	1.68*	1.04
CHC_T00005038001	CcGal-Sulfurylase II	0.18	0.18	0.63	−0.29	1.25	1.54*
CHC_T00008027001	CcGal-Sulfurylase II	1624.38	106.60	4.46	−4.27*	−9.09*	−4.82*
CHC_T00008308001	CcGal-Sulfurylase II	0.00	0.09	1.28	2.75	6.3*	3.58
CHC_T00008371001	CcGal-Sulfurylase II	0.15	0.08	229.98	−1.04	10.07*	11.11*
CHC_T00008447001	CcGal-Sulfurylase II	0.34	0.23	149.37	−0.70	8.31*	9.04*
CHC_T00008940001	CcGal-Sulfurylase II	0.17	0.25	267.11	0.26	10.03*	9.78*
CHC_T00009021001	CcGal-Sulfurylase II	0.34	0.23	798.08	−0.70	10.71*	11.48*
CHC_T00009181001	CcGal-Sulfurylase II	0.00	3.92	82.11	9.38	13.38*	4.03*
CHC_T00009289001	CcGal-Sulfurylase II	0.10	0.18	224.01	0.51	10.66*	10.14*
CHC_T00009416001	CcGal-Sulfurylase II	0.15	2.37	332.49	3.88	10.64*	6.8*
CHC_T00009440001	CcGal-Sulfurylase II	4.02	6.26	16.39	0.34	1.51*	1.17
CHC_G00008501001	CcGal-Sulfurylase II	0.15	0.18	0.57	0.06	1.45	1.39
CHC_G00008516001	CcGal-Sulfurylase II	1,204.35	2,237.10	1,467.91	0.64	−0.24	−0.87
CHC_T00009418001	CcGal-Sulfurylase II	0.12	0.86	143.02	2.70	9.72*	7.04*
CHC_G00009027001	CcGH16-1	0.20	0.13	0.13	−0.85	−1.12	−0.27
CHC_G00009399001	CcGH16-2	38.71	72.13	73.21	0.67	0.39	−0.26
CHC_T00009578001	CcGH16-3	13.49	80.59	102.88	2.31*	2.43*	0.13
CHC_T00000714001	CcGT14-1	1.51	4.62	8.94	1.35	2.09*	0.74
CHC_G00005911001	CcGT14-2	60.71	56.88	63.06	−0.33	−0.48	−0.14
CHC_T00008436001	CcGT14-3	3.89	12.21	55.43	1.29	3.32*	2.03
CHC_T00008553001	CcGT14-4	24.81	19.25	9.61	−0.68	−1.86*	−1.18
CHC_T00008814001	CcGT14-5	4.72	7.42	14.17	0.40	1.05*	0.65
CHC_G00009017001	CcGT14-6	0.00	0.00	0.00	NA	NA	NA
CHC_G00008920001	CcGT47-1	18.13	33.17	28.20	0.70	0.12	−0.56
CHC_T00009415001	CcGT47-2	11.85	10.34	6.08	−0.55	−1.47*	−0.93
CHC_T00009549001	CcGT47-3	5.31	13.73	394.05	1.19	5.72*	4.54*
CHC_T00001315001	CcGT7-1	3.28	13.38	134.67	1.86	4.85*	3.01*
CHC_T00008612001	CcGT7-2	14.28	14.83	70.89	−0.18	1.79*	1.97*
CHC_G00008980001	CcGT7-3	0.00	0.00	0.00	NA	NA	NA
CHC_G00009035001	CcGT7-4	37.82	30.46	29.12	−0.55	−0.93	−0.37
CHC_T00008342001	CcST2-1	293.14	305.39	77.18	−0.14	−2.45*	−2.29*
CHC_T00008402001	CcST2-2	4.30	0.57	166.00	−3.05	4.78*	7.85*
CHC_G00008775001	CcST2-3	1.22	0.97	2.35	−0.64	0.41	1.04
CHC_T00008834001	CcST2-4	0.14	3.05	200.87	4.27	10.01*	5.68*
CHC_T00008846001	CcST2-5	363.00	264.18	84.62	−0.66	−2.63*	−1.96*
CHC_T00009000001	CcST2-6	0.87	25.16	76.38	4.75	5.96*	1.24
CHC_G00009100001	CcST2-7	7.17	6.04	21.12	−0.55	1.06	1.61

**Figure 5:**
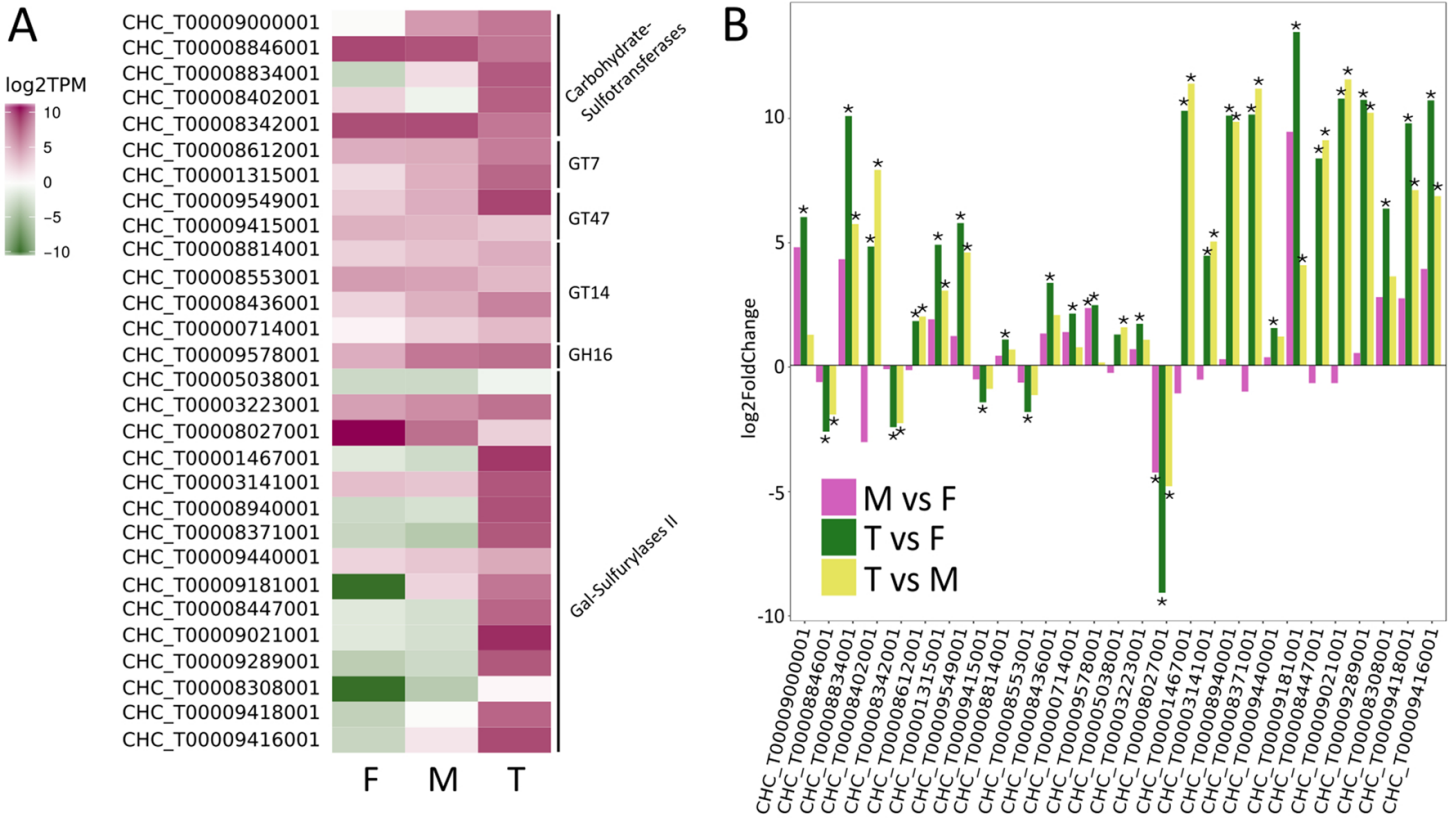
(**A**) Heatmap showing gene expression levels (log2 TPM) of the differentially regulated carrageenan-biosynthesis genes of interest. (**B**) Differential expression levels of these genes of interest in three comparisons, M v F, F v T, and M v T; M-male gametophytes, F-female gametophytes, T- tetrasporophytes. Significant differences are marked with an asterisk.

Only one galactose-sulfurylase II gene was significantly upregulated in gametophytes relative to tetrasporophytes (Fig. 5, Table 4, CHC_T00008027001). The same galactose-sulfurylase also showed a significant difference in gene expression between the male and female gametophytes. This enzyme is interesting as it is phylogenetically distant in the galactose-sulfurylase II phylogenetic tree (Fig. 4) suggesting a difference in specificity from the originally identified galactose-sulfurylase II enzymes.

Tetrasporophytes have primarily lambda-carrageenan in their ECM which is depleted in 3,6-anhydro-bridges^15–18^. Unexpectedly, 10 galactose-sulfurylase II genes, encoding enzymes involved in the formation of 3,6-anhydro-bridges^7^, were upregulated in tetrasporophytes relative to the male and female gametophytes. Natural carrageenans are hybrid polymers of different carrabiose repeating units. It is tempting to imagine that the sulfurylase genes expressed during the tetrasporophyte stage are for the formation of highly specialized glycan sequences containing the 3,6-anhydro-D-galactose motif within the lambda carrageenan backbone that forms the mainstay of the carrageenan chains in the tetrasporophytes^17^. These 3,6-anhydro-bridge motifs would induce structural changes in the carrageenan architecture and the specialized carrageenan sequence motifs might also be influential in recognition, signalling or developmental events.

The galactose-sulfurylases are one of red algae’s most interesting, yet least understood, family of enzymes and the differential expression of their genes between the red algal life stages underscores the importance of this multigenic family to ECM modification in red algae. More biochemical studies are required to fully understand the fine specificities and unknown catalytic mechanism of this unique enzyme family and to confirm that other members of the family are also involved in 3,6-anhydro-bridge formation.

#### Glycosyl Transferases (GTs)

Unlike land plants, sulfated polysaccharides dominate the extracellular matrices of algae. The other main well of natural sulfated polysaccharides comes from animals; they have a special subset of charged polysaccharides called the glycosaminoglycans or GAGs. Structurally the heterogeneous GAGs are similar to carrageenan, they are long, unbranched, often sulfated, and consist of a repeating disaccharide unit. Many useful and interesting parallels in carrageenan biosynthesis can be extrapolated from the synthesis of animal GAGs^52,53^. Both chondroitin sulfate and heparan sulfate synthesis begin with the stepwise addition of monosaccharides by different glycosyl transferases (GTs) to form a tetrasaccharide linker attached to a core protein. Following linker region synthesis, specific GTs successively add monosaccharides to form the repetitive disaccharide backbone polymer of the GAG. Sulfotransferases (STs) act on the growing polysaccharide chain in a regiospecific manner. Further modifications by other enzymes are also possible.

Several genes, encoding glycosyl transferase enzymes from different CAZy families^54^, were differentially regulated between the life cycle stages of *Chondrus*. Through manual gene annotation we identified specific GT families we hypothesize to be involved in carrageenan glycosidic bond formation.

*Chondrus* has four GT7 enzymes whereas the red macroalga *Porphyra umbilicalis*, which produces the sulfated polysaccharide porphyran in its ECM and whose carbohydrate-active enzymes have been annotated^55^, has only one. The closest non-algal homologues of the GT7s are found in animals^27^ and are implicated in GAG biosynthesis, particularly chondroitin sulfate biosynthesis as beta-1,4-*N*-acetylgalactosaminyltransferases^53^. The relationship to sulfated polysaccharide biosynthesis in animals suggests the red algal GT7 members may also be involved in sulfated carbohydrate biosynthesis and that at least some of the actors involved in sulfated polysaccharide biosynthesis have evolved from the last eukaryotic common ancestor^27,40^. Based on the activities already described in the GT7 family^54^, the GT7 enzymes from *Chondrus* are predicted to transfer an activated galactose moiety to the growing carrageenan chain to form the beta-1,4-linkage. Two GT7 encoding genes were upregulated in *Chondrus* tetrasporophytes compared to the gametophytes (Table 4, Fig. 5).

The three GT47 enzymes from *Chondrus* are most closely related to plant GT47 members involved in cell wall biosynthesis. Activities described in plants include xyloglucan beta-galactosyltransferase (hemicellulose biosynthesis)^56^, pectin beta-glucuronyltransferase^57^, pectin xylogalacturonan xylosyltransferase^58^ and arabinan alpha-L-arabinosyltransferase (pectic arabinan biosynthesis)^59^. GT47 members are also involved in heparan sulfate biosynthesis in animals, adding beta-1,4-glucuronic acid to the growing heparan chain^53^. The relationship to other GT47 enzymes involved in the biosynthesis of negatively charged (pectin and heparan sulfate) and sulfated (heparan sulfate) extracellular glycans leads to the prediction that these enzymes are implicated in carrageenan chain elongation in *Chondrus*. It is difficult to assign a more specific annotation based on the diverse activities already described in the GT47 family. There is one GT47 member from *Chondrus* that is significantly upregulated (Table 4, Fig. 5) in tetrasporophytes relative to gametophytes (CHC_T00009549001). The GT47 gene CHC_T00009415001 shows higher expression levels in the gametophytes which is significant in the female gametophytes relative to the tetrasporophytes (Table 4, Fig. 5).

There are six *Chondrus* GT14 enzymes and four *Porphyra* GT14 enzymes that find their closest non-algal homologues in plant GT14 enzymes. Three *Arabidopsis thaliana* enzymes have been characterized from plants and all are implicated in type II arabinogalactan biosynthesis in the cell wall, acting as β-1,6-glucuronosyltransferases^60,61^, transferring a negatively charged glucuronic acid molecule to galactan oligosaccharides. Metazoan GT14 enzymes have demonstrated β-1,6-*N*-acetylglucosaminyltransferase activity, forming crucial side chain branches in core 2 mucin O-glycans^62^ and the blood group I-antigen during embryonic development^63^, as well as peptide O-β-xylosyltransferase activity, essential as the first step in the biosynthesis of chondroitin, dermatan and heparan sulfate^64^. Involvement of the GT14 family in negatively charged and sulfated glycan biosynthesis suggests that the *Chondrus* enzymes are implicated in carrageenan biosynthesis and that the *Porphyra* enzymes are implicated in porphyran biosynthesis. It is difficult to predict a specific activity though the *Chondrus* GT14 enzymes may be involved in chain initiation or elongation in carrageenan biosynthesis. There are three GT14 members that have higher gene expression in tetrasporophytes relative to gametophytes (Table 4, Fig. 5). Conversely, one GT14 is upregulated in the gametophytes which is significant for the female relative to the tetrasporophytes (Table 4, Fig. 5). In both plants and animals, the GT14 enzymes have demonstrated important roles in development and this might hold true in red algae as well.

#### Carbohydrate-Sulfotransferases

Carboydrate sulfotransfereases (STs) act by adding sulfonyl groups from 3′phosphoadenosine-5′phosphosulfate (PAPS) to a glycoside receptor^65^. *Chondrus* has seven predicted carbohydrate-STs with their non-algal ST homologues found in animals and active in GAG sulfations. Comparatively, *Porphyra* has only one carbohydrate-ST, likely active as an L-galactose-6-O-sulfotransferase in the biosynthesis of porphyran^55^. Brown algal carbohydrate-STs also share an evolutionary relationship with animal STs involved in GAG biosynthesis^66^. Land plants, which don’t grow in a saline environment, aren’t known to have sulfated polysaccharides and lack the STs common to animals and algae. Sea plants, such as the seagrass *Zostera marina*^67^, have regained capability to sulfate their polysaccharides through unknown mechanisms. Overall, the evolutionary relationship between animal carbohydrate-STs and algal STs lends support to the ancestral nature of sulfated polysaccharide biosynthesis in eukaryotes.

Carrageenan is the only known sulfated carbohydrate in *Chondrus*, thus it remains the prime substrate candidate for the carbohydrate-ST family of enzymes^65^. Carbohydrate-STs tailor the physico-chemical and mechanical properties of the algal ECM providing both elasticity and strength which intuitively would help protect against the strong ocean currents^68^. In animals, diverse regiospecific sulfation patterns on GAGs are both structural and a source of extracellular biological information to cells, influencing recognition events, signalling pathways and developmental processes^65,69,70^. In analogy to the animal GAGs^69,70^, these specialized carrageenan sulfations sequence motifs may also be important for recognition, signalling or development events in algae. Unlike animals, sulfatases have not yet conclusively been identified or characterized in red algae. It is unknown how red algae cope with ECM remodeling in the absence of known sulfatases, though the red algal microbiome has a role in the recycling of sulfur as it produces sulfatases specific for carrageenan^71–74^.

Altogether, five carbohydrate-ST enzymes were differentially regulated between the *Chondrus* life cycle stages (Table 4, Fig. 5). Four of the carbohydrate-ST genes were differentially expressed between the gametophytes and the tetrasporophytes. This suggests major differences in sulfation patterns between the gametophyte and tetrasporophyte life stages, which is supported in the literature^15–18^.

#### Glycoside hydrolases

Glycoside hydrolases are enzymes that hydrolyze the glycosidic bond through the addition of a water molecule and in some specialized instances they may act as transglycosidases, forming a glycosidic bond. *Chondrus* has three genes encoding family 16 glycoside hydrolases (GH16). A phylogeny on the GH16 family has been previously undertaken and the three GH16 members from *Chondrus* (CcGH16-1, CcGH16-2 and CcGH16-3) are found in the same clade as bacterial agarases, porphyranases and three red algal enzymes from *Porphyra*^55^. Red algae likely obtained these genes through a horizontal gene transfer event between marine bacteria and red algae^55^*.* Since agar and porphyran are not present in the ECM of *Chondrus*, the function of the *Chondrus* GH16 enzymes may be in the remodeling of its own sulfated ECM polysaccharide, carrageenan. The gene expression for one family 16 glycoside hydrolase was upregulated both in male gametophytes and tetrasporophytes relative to the female gametophytes (Table 4, Fig. 5). This interesting result suggests molecular differences in carrageenan structure between male and female gametophytes.

## Conclusion

Carrageenan biosynthesis between the life cycle phases implicates the differential regulation of several rare multigenic gene families in *Chondrus*. Carrageenan can, at times, make up over 50% of the dry weight of *Chondrus*^18^, highlighting the importance of the carrageenan macromolecule to the success of this red alga. Here, we demonstrate that carrageenan biosynthesis in these multigenic families is at least partially regulated through modulation of gene transcription between the life cycles in *Chondrus,* indicating a level of genetic control. This genetic control is likely at least partially responsible for the molecular differences in carrageenan structure between the tetrasporophyte and gametophyte isomorphic life cycle stages.

The foremost differences in carrageenan-related gene expression between life cycle stages is seen between the gametophytes and the tetrasporophytes. Thus, the differential regulation of the carrageenan-utilization genes between life stages may be important for the biosynthesis of specialized glycan motifs within the polymer. Only two genes, CcGH16-3 and a galactose-sulfurylase II (CHC_T00008027001), showed differential gene expression between the male and female gametophytes. Male and female gametophytes have been described as having similar carrageenan structures; however, these differential gene expression results suggest some differences in architecture. This also supports data from infection experiments with the pathogen *Ulvella operculata* in which male gametophytes were found to be slightly more infected than female gametophytes, though never to the level of tetrasporophytes^23^. Male gametophytes shed their apices following spermatial release^35^ and generally look more tattered than the female gametophytes (SA Krueger-Hadfield, personal observation). No studies have explored in detail the male gametophytes in natural populations of *Chondrus*. Differences in carrageenan structure may manifest at the population level though more work is necessary to link these data to field-patterns in ploidy and sex ratios. For example, if there are differences between male gametophyte, female gametophyte, and tetrasporophtye infection rates linked to carrageenan content, as has been suggested^6,23^, then there may be corresponding differences in abundances of thalli in the field due in part to infection-induced dislodgement. Not only do our data support the hypothesis of niche partitioning between *Chondrus* life cycle stages, by providing evidence of differential gene expression governing the physiological state of gametophytes and tetrasporophytes^32^, they pose excellent topics for further investigation. Future studies, exploring niche differentiation and underlying genetic components, could shed light on the maintenance of haplodiplontic life cycles and contribute to understanding the concept of the niche that is relevant for the organism in question.

Altogether, these data strongly support that macromolecular structure of carrageenan in the ECM of *Chondrus* correlates to the physiological differentiation of the alga throughout the isomorphic life stages.

## Supplementary information

Supplementary File 1.

Supplementary Fig. 1.

## Abbreviations

ECM: Extracellular matrix
GAG: Glycosaminoglycan
GH: Glycoside hydrolase
GT: Glycosyl transferase
ST: Sulfotransferase

## References

[1] Butterfield N (2000). *Bangiomorpha pubescens* n. gen., n. sp.: implications for the evolution of sex, multicellularity, and the Mesoproterozoic/Neoproterozoic radiation of eukaryotes. Paleobiology.

[2] Gibson TM (2018). Precise age of *Bangiomorpha pubescens* dates the origin of eukaryotic photosynthesis. Geology.

[3] Bengtson S, Sallstedt T, Belivanova V, Whitehouse M (2017). Three-dimensional preservation of cellular and subcellular structures suggests 1.6 billion-year-old crown-group red algae. PLoS Biol..

[4] Popper ZA (2011). Evolution and diversity of plant cell walls: from algae to flowering plants. Annu. Rev. Plant Biol..

[5] Potin P, Bouarab K, Kupper F, Kloareg B (1999). Oligosaccharide recognition signals and defence reactions in marine plant-microbe interactions. Curr. Opin. Microbiol..

[6] Bouarab K, Potin P, Correa J, Kloareg B (1999). Sulfated oligosaccharides mediate the interaction between a marine red alga and its green algal pathogenic endophyte. Plant Cell.

[7] Genicot-Joncour S (2009). The cyclization of the 3,6-anhydro-galactose ring of iota-carrageenan is catalyzed by two D-galactose-2,6-sulfurylases in the red alga *Chondrus crispus*. Plant Physiol..

[8] Hehemann JH (2010). Transfer of carbohydrate-active enzymes from marine bacteria to Japanese gut microbiota. Nature.

[9] Ciancia M, Matulewicz MC, Cerezo AS (1997). A L-galactose-containing carrageenan from cystocarpic *Gigartina skottsbergii*. Phytochemistry.

[10] Stortz CA, Cases MR, Cerezo AS (1997). The system of agaroids and carrageenans from the soluble fraction of the tetrasporic stage of the red seaweed *Iridaea undulosa*. Carbohyd. Polym..

[11] Takano, R., Shiomoto, K., Kamei, K., Hara, S., & Hirase, S (2003) Occurrence of carrageenan structure in an agar from the red seaweed *Digenea simplex* (Wulfen) *C. agardh* (Rhodomelaceae, Ceramiales) with a short review of carrageenan-agarocolloid hybrid in the florideophycidae. *Bot. Mar.* **46**, 142–150. 10.1515/Bot.2003.015.

[12] Navarro DA, Stortz CA (2003). Determination of the configuration of 3,6-anhydrogalactose and cyclizable alpha-galactose 6-sulfate units in red seaweed galactans. Carbohydr. Res..

[13] Chen LC-M, Mclachla J (1972). Life history of *Chondrus crispus* in culture. Can. J. Bot..

[14] Krueger-Hadfield SA, Collen J, Daguin-Thiebaut C, Valero M (2011). Genetic population structure and mating system in *Chondrus crispus* (Rhodophyta). J. Phycol..

[15] Fournet I, Deslandes E, Floch JY (1993). Iridescence - a useful criterion to sort gametophytes from sporophytes in the red alga *Chondrus crispus*. J. Appl. Phycol..

[16] Chen LCM, Mclachla J, Neish AC, Shacklock PF (1973). Ratio of kappa-carrageenan to lambda-carrageenan in nuclear phases of Rhodophycean algae, *Chondrus crispus* and *Gigartina stellata*. J. Mar. Biol. Assoc. UK.

[17] McCandless E, Craigie J, Walter J (1973). Carrageenans in the gametophytic and sporophytic stages of *Chondrus crispus*. Planta.

[18] Pereira L (2013). Population studies and carrageenan properties in eight Gigartinales (Rhodophyta) from Western Coast of Portugal. Sci. World J.

[19] Chopin T, Floc'h J-Y (1992). Eco-physiological and biochemical study of two of the most contrasting forms of *Chondrus crispus* (Rhodophyta, Gigartinales). Mar. Ecol. Prog. Ser..

[20] Tasende MG, Cid M, Fraga MI (2012). Spatial and temporal variations of *Chondrus crispus* (Gigartinaceae, Rhodophyta) carrageenan content in natural populations from Galicia (NW Spain). J. Appl. Phycol..

[21] Collen J (2014). *Chondrus crispus* - A present and historical model organism for red seaweeds. Adv. Bot. Res..

[22] Correa JA, Mclachlan JL (1991). Endophytic algae of *Chondrus crispus* (Rhodophyta). 3. Host specificity. J. Phycol..

[23] Krueger-Hadfield, S. A. Population structure in the haploid-diploid red alga *Chondrus crispus*: mating system, genetic differentiation and epidemiology. Doctoral thesis, UPMC Paris 6 with l'Universidad católica de Chile (2011).

[24] Destombe C, Valero M, Vernet P, Couvet D (1989). What controls haploid–diploid ratio in the red alga *Gracilaria verrucosa*. . J. Evol. Biol..

[25] Thornber CS, Gaines SD (2004). Population demographics in species with biphasic life cycles. Ecology.

[26] Craigie, J. & Wong, H. Carrageenan biosynthesis. *Proceedings of the International Seaweed Symposium*, 369–377 (1979).

[27] Ficko-Blean E, Hervé C, Michel G (2015). Sweet and sour sugars from the sea: the biosynthesis and remodeling of sulfated cell wall polysaccharides from marine macroalgae. PiP.

[28] Wong KF, Craigie JS (1978). Sulfohydrolase activity and carrageenan biosynthesis in *Chondrus crispus* (Rhodophyceae). Plant Physiol..

[29] van de Velde F, Knutsen SH, Usov AI, Rollema HS, Cerezo AS (2002). H-1 and C-13 high resolution NMR spectroscopy of carrageenans: application in research and industry. Trends Food Sci. Tech..

[30] Campo VL, Kawano DF, Silva DBJ, Carvalho I (2009). Carrageenans: biological properties, chemical modifications and structural analysis - a review. Carbohyd. Polym..

[31] Carrington E, Grace SP, Chopin T (2001). Life history phases and the biomechanical properties of the red alga *Chondrus crispus* (Rhodophyta). J. Phycol..

[32] Hughes JS, Otto SP (1999). Ecology and the evolution of biphasic life cycles. Am. Nat..

[33] Krueger-Hadfield SA (2019). What's ploidy got to do with it? Understanding the evolutionary ecology of macroalgal invasions necessitates incorporating life cycle complexity. Evol. Appl..

[34] Garbary DJ, Tompkins E, White K, Corey P, Kim JK (2011). Temporal and spatial variation in the distribution of life history phases of *Chondrus crispus* (Gigartinales, Rhodophyta). Algae.

[35] Tveter-Gallagher E, Mathieson AC, Cheney DP (1980). Ecology and developmental morphology of male plants of *Chondrus crispus* (Gigartinales, Rhodophyta). J. Phycol..

[36] Krueger-Hadfield SA, Roze D, Mauger S, Valero M (2013). Intergametophytic selfing and microgeographic genetic structure shape populations of the intertidal red seaweed *Chondrus crispus*. Mol. Ecol..

[37] Yaphe W, Arsenault GP (1965). Improved resorcinol reagent for determination of fructose and of 3,6-anhydrogalactose in polysaccharides. Anal. Biochem..

[38] Dyck L, De Wreede RE, Garbary D (1985). Life history phases in *Iridaea cordata* (Gigartinaceae): relative abundance and distribution from British Columbia to California. Jap. J. Phycol..

[39] Bolger AM, Lohse M, Usadel B (2014). Trimmomatic: a flexible trimmer for Illumina sequence data. Bioinformatics.

[40] Collen J (2013). Genome structure and metabolic features in the red seaweed *Chondrus crispus* shed light on evolution of the Archaeplastida. Proc. Natl. Acad. Sci. USA.

[41] Haas BJ (2003). Improving the *Arabidopsis* genome annotation using maximal transcript alignment assemblies. Nucl. Acids Res..

[42] Bray NL, Pimentel H, Melsted P, Pachter L (2016). Near-optimal probabilistic RNA-seq quantification. Nat. Biotechnol..

[43] Love MI, Huber W, Anders S (2014). Moderated estimation of fold change and dispersion for RNA-seq data with DESeq2. Genome Biol..

[44] Madden TL, Tatusov RL, Zhang J (1996). Applications of network BLAST server. Methods Enzymol..

[45] Mitchell A (2015). The InterPro protein families database: the classification resource after 15 years. Nucl. Acids Res..

[46] Punta M (2012). The Pfam protein families database. Nucl. Acids Res..

[47] Altschul SF, Gish W, Miller W, Myers EW, Lipman DJ (1990). Basic local alignment search tool. J. Mol. Biol..

[48] Katoh K, Standley DM (2013). MAFFT multiple sequence alignment software version 7: improvements in performance and usability. Mol. Biol. Evol..

[49] Hall TA (1998). BioEdit: a user-friendly biological sequence alignment editor and analysis program for Windows 95/98/NT. Nucl. Acids. Symp. Ser..

[50] Whelan S, Goldman N (2001). A general empirical model of protein evolution derived from multiple protein families using a maximum-likelihood approach. Mol. Biol. Evol..

[51] Kumar S, Stecher G, Li M, Knyaz C, Tamura K (2018). MEGA X: molecular evolutionary genetics analysis across computing platforms. Mol. Biol. Evol..

[52] Kusche-Gullberg M, Kjellen L (2003). Sulfotransferases in glycosaminoglycan biosynthesis. Curr. Opin. Struct. Biol..

[53] Breton C, Fournel-Gigleux S, Palcic MM (2012). Recent structures, evolution and mechanisms of glycosyltransferases. Curr. Opin. Struct. Biol..

[54] Lombard V, Golaconda Ramulu H, Drula E, Coutinho PM, Henrissat B (2014). The carbohydrate-active enzymes database (CAZy) in 2013. Nucl. Acids Res..

[55] Brawley SH (2017). Insights into the red algae and eukaryotic evolution from the genome of *Porphyra umbilicalis* (Bangiophyceae, Rhodophyta). Proc. Natl. Acad. Sci. U.S.A..

[56] Madson M (2003). The MUR3 gene of *Arabidopsis* encodes a xyloglucan galactosyltransferase that is evolutionarily related to animal exostosins. Plant Cell.

[57] Iwai H, Masaoka N, Ishii T, Satoh S (2002). A pectin glucuronyltransferase gene is essential for intercellular attachment in the plant meristem. Proc. Natl. Acad. Sci. USA.

[58] Jensen JK (2008). Identification of a xylogalacturonan xylosyltransferase involved in pectin biosynthesis in *Arabidopsis*. Plant Cell.

[59] Harholt J (2006). ARABINAN DEFICIENT 1 is a putative arabinosyltransferase involved in biosynthesis of pectic arabinan in *Arabidopsis*. Plant Physiol..

[60] Dilokpimol A, Geshi N (2014). *Arabidopsis thaliana* glucuronosyltransferase in family GT14. Plant Signal. Behav..

[61] Knoch E (2013). A beta-glucuronosyltransferase from *Arabidopsis thaliana* involved in biosynthesis of type II arabinogalactan has a role in cell elongation during seedling growth. Plant J..

[62] Pak JE (2006). X-ray crystal structure of leukocyte type core 2 beta 1,6-N-acetylglucosaminyltransferase—evidence for a convergence of metal ion-independent glycosyltransferase mechanism. J. Biol. Chem..

[63] Bierhuizen MFA, Mattei MG, Fukuda M (1993). Expression of the developmental-I antigen by a cloned human cDNA-encoding a member of a beta-1,6-*N*-acetylglucosaminyltransferase gene family. Gene Dev..

[64] Wilson IBH (2004). The never-ending story of peptide O-xylosyltransferase. Cell. Mol. Life Sci..

[65] Bowman KG, Bertozzi CR (1999). Carbohydrate sulfotransferases: mediators of extracellular communication. Chem. Biol..

[66] Michel G, Tonon T, Scornet D, Cock JM, Kloareg B (2010). The cell wall polysaccharide metabolism of the brown alga *Ectocarpus siliculosus*. Insights into the evolution of extracellular matrix polysaccharides in Eukaryotes. New Phytol..

[67] Olsen JL (2016). The genome of the seagrass *Zostera marina* reveals angiosperm adaptation to the sea. Nature.

[68] Kloareg B, Quatrano RS (1988). Structure of the cell walls of marine algae and ecophysiological functions of the matrix polysaccharides. Oceanogr. Mar. Biol. Annu. Rev..

[69] Hayes A (2018). Biodiversity of CS-proteoglycan sulphation motifs: chemical messenger recognition modules with roles in information transfer, control of cellular behaviour and tissue morphogenesis. Biochem. J..

[70] Esko JD, Selleck SB (2002). Order out of chaos: Assembly of ligand binding sites in heparan sulfate. Annu. Rev. Biochem..

[71] Ficko-Blean E (2017). Carrageenan catabolism is encoded by a complex regulon in marine heterotrophic bacteria. Nat. Commun..

[72] Prechoux A, Genicot S, Rogniaux H, Helbert W (2013). Controlling carrageenan structure using a novel formylglycine-dependent sulfatase, an endo-4S-iota-carrageenan sulfatase. Mar. Biotechnol. (NY).

[73] Hettle AG (2019). Insights into the kappa/iota-carrageenan metabolism pathway of some marine *Pseudoalteromonas* species. Commun. Biol..

[74] Genicot S (2014). Discovery of a novel iota carrageenan sulfatase isolated from the marine bacterium *Pseudoalteromonas carrageenovora*. Front. Chem..

