# Supplementary material for: To gel or not to gel: differential expression of carrageenan-related genes between the gametophyte and tetasporophyte life cycle stages of the red alga Chondrus crispus
Source: Sci Rep. 2020 Jul 13;10:11498. doi: 10.1038/s41598-020-67728-6 (PMC7359372; doi:10.1038/s41598-020-67728-6)
Supplement: Supplementary file 2 — Supplementary Fig. 1. [file 41598_2020_67728_MOESM2_ESM.pdf]

To gel or not to gel: Differential expression of carrageenan-related genes between the gametophyte and tetasporophyte life cycle stages of the red alga *Chondrus crispus*

Author List:

*Agnieszka Lipinska, Station Biologique de Roscoff, UMR8227, Laboratory of Integrative Biology of Marine Models, CNRS and Sorbonne University,Place George Teissier, Roscoff 29688, France. Email: alipinska@sb-roscoff.fr.

Jonas Collen, Station Biologique de Roscoff, UMR8227, Laboratory of Integrative Biology of Marine Models, CNRS and Sorbonne University,Place George Teissier, Roscoff 29688, France

Stacy A. Krueger-Hadfield, Department of Biology, The University of Alabama at Birmingham, Campbell Hall 464, 1300 University Blvd, Birmingham, AL 35294, USA.

Theo Mora, Station Biologique de Roscoff, UMR8227, Laboratory of Integrative Biology of Marine Models, CNRS and Sorbonne University,Place George Teissier, Roscoff 29688, France.

*Elizabeth Ficko-Blean, Station Biologique de Roscoff, UMR8227, Laboratory of Integrative Biology of Marine Models, CNRS and Sorbonne University,Place George Teissier, Roscoff 29688, France. Email: efickoblean@sb-roscoff.fr.

*Corresponding Author

Supplementary Figure 1. Resorcinol test results. All individuals used in the analyses underwent the resorcinol test for colourimetric prediction of life cycle stage37,38. A strong red colour identifies Chondrus gametophytes and light pink identifies tetrasporophytes. T- represents tetrasporophytes, M-male gametophytes, F-female gametophytes and GS is the genome strain.

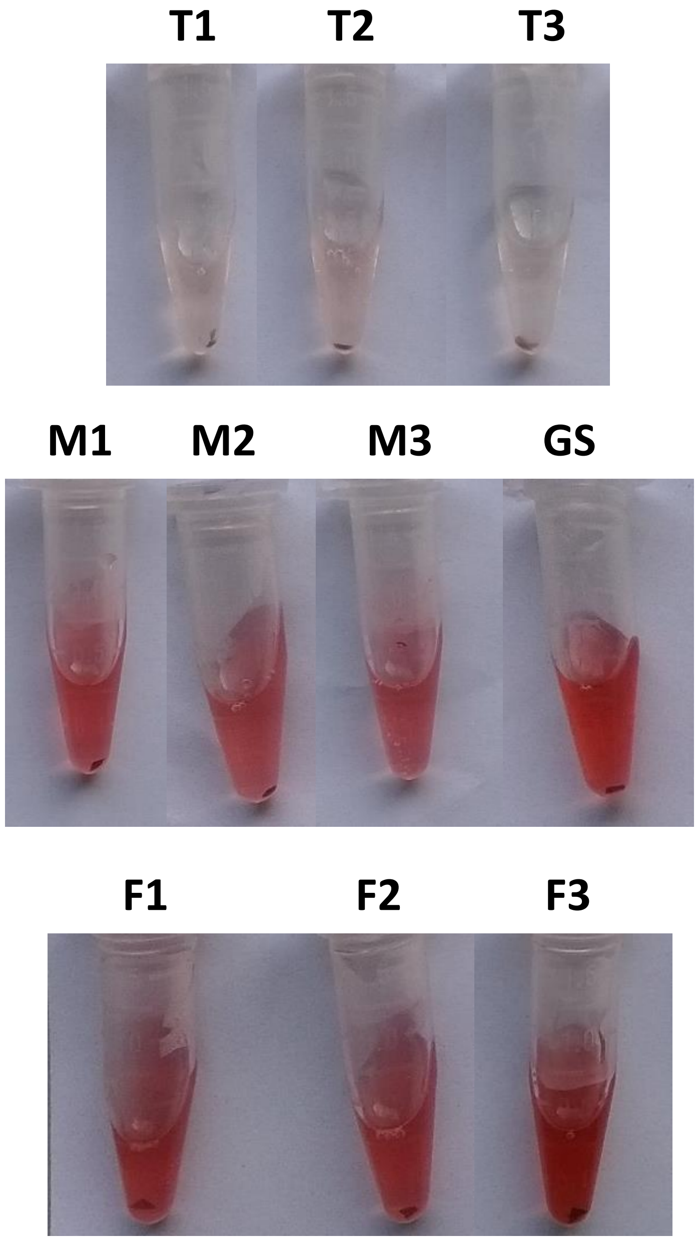
T1
T2
T3
M1
M2
M3
GS
F1
F2
F3
